# Whole genome association mapping by incompatibilities and local perfect phylogenies

**DOI:** 10.1186/1471-2105-7-454

**Published:** 2006-10-16

**Authors:** Thomas Mailund, Søren Besenbacher, Mikkel H Schierup

**Affiliations:** 1 Department of Statistics, University of Oxford, UK; 2 Bioinformatics Research Center, University of Aarhus, Denmark

## Abstract

**Background:**

With current technology, vast amounts of data can be cheaply and efficiently produced in association studies, and to prevent data analysis to become the bottleneck of studies, fast and efficient analysis methods that scale to such data set sizes must be developed.

**Results:**

We present a fast method for accurate localisation of disease causing variants in high density case-control association mapping experiments with large numbers of cases and controls. The method searches for significant clustering of case chromosomes in the "perfect" phylogenetic tree defined by the largest region around each marker that is compatible with a single phylogenetic tree. This perfect phylogenetic tree is treated as a decision tree for determining disease status, and scored by its accuracy as a decision tree. The rationale for this is that the perfect phylogeny near a disease affecting mutation should provide more information about the affected/unaffected classification than random trees. If regions of compatibility contain few markers, due to e.g. large marker spacing, the algorithm can allow the inclusion of incompatibility markers in order to enlarge the regions prior to estimating their phylogeny. Haplotype data and phased genotype data can be analysed. The power and efficiency of the method is investigated on 1) simulated genotype data under different models of disease determination 2) artificial data sets created from the HapMap ressource, and 3) data sets used for testing of other methods in order to compare with these. Our method has the same accuracy as single marker association (SMA) in the simplest case of a single disease causing mutation and a constant recombination rate. However, when it comes to more complex scenarios of mutation heterogeneity and more complex haplotype structure such as found in the HapMap data our method outperforms SMA as well as other fast, data mining approaches such as HapMiner and Haplotype Pattern Mining (HPM) despite being significantly faster. For unphased genotype data, an initial step of estimating the phase only slightly decreases the power of the method. The method was also found to accurately localise the known susceptibility variants in an empirical data set – the ΔF508 mutation for cystic fibrosis – where the susceptibility variant is already known – and to find significant signals for association between the CYP2D6 gene and poor drug metabolism, although for this dataset the highest association score is about 60 kb from the CYP2D6 gene.

**Conclusion:**

Our method has been implemented in the Blossoc (BLOck aSSOCiation) software. Using Blossoc, genome wide chip-based surveys of 3 million SNPs in 1000 cases and 1000 controls can be analysed in less than two CPU hours.

## Background

With the publication of the human HapMap, phase I [1], and the completion of genotyping for phase II, association mapping is entering a new era. Whole genome scans using 317 K or 500 K SNP chips, currently available [2], and their higher density descendants, will be performed in large sets of cases and controls in a search for high-frequency, low penetrance variants that increases susceptibility to common, complex diseases [3,4]. Recently published genome scans in very large cohorts have yielded such variants that show reproducible effects [5-7]. Knowledge about these variants is expected to lead to a better understanding of disease initiation and progression, to identify pharmacological targets for prevention of the disease in high risk individuals as well as individually based treatment. Case-control association mapping allows zooming in on relatively smaller regions than linkage mapping in pedigrees because recombination events in the (deep) unknown genealogy of the sample have decoupled all but the closest markers from the (unknown) susceptibility variants. Furthermore, it is usually easier to recruit large numbers of unrelated individuals for these studies.

With the immense data generation (a single data set readily contains more than 1 billion genotypes), there is an evident need for efficient methods for localisation of susceptibility variants which are also very fast and which can guide the subsequent selection of further markers in two- or multi-stage designs [8,9]. Many studies resort to a marker by marker approach, i.e. single marker tests for independence between cases and controls, typically by a 2 × 2 (allelic) or 2 × 3 (genotypic) Fisher's exact test or *χ*^2^-test. The best set of markers (and markers close to these, i.e. in strong linkage disequilibrium) will then be selected for scrutiny in a larger set of individuals or a different population for replication of the association. However, unless the susceptibility variant is included among the markers typed, a marker by marker approach does not seem efficient [10] since it disregards the dependency of close markers caused by their sharing of common genealogical history, a sharing that decreases with the level of recombination between the markers. For instance, if a strongly associated marker is flanked to the right by another associated marker (and not to the left), then one would expect a higher probability for the true location slightly to the right of the marker than slightly to the left.

A popular alternative to single marker association is to (approximately) model the whole data generating process. In this case this is termed the *ancestral recombination graph *(ARG) [11]. This has most often been done by treating the disease locus as an unknown parameter, whose posterior distribution is estimated from genotype data and an underlying model of the process that generated the data. The posterior is usually numerically evaluated through Monte Carlo methods such as *importance sampling *[12] and *Markov Chain Monte Carlo *(MCMC) integration [13,14]. For the mathematical model of the underlying process, the complexity varies from assuming no relationship among diseased individuals except through the original mutation of the disease gene (a star topology of cases) [15,16], through coalescent theory based methods where either a tree [13,14] or an ancestral recombinaion graph is modelled [12]. These models have shown to be accurate on small data sets [17,18], but so far they are not able to treat much more than hundreds of cases and controls and hundreds of markers in a reasonable time.

Furthermore, being based on very specific models, these methods are also sensitive to a bad fit to the model, which may well be the case for the human genome where recombination hotspots seem prevalent as do signs of recent population growth, and where the ascertainment biases introduced by the choice of markers are generally unknown and therefore difficult to control for [19].

Thus, there is a need for more sophisticated methods than single marker associations; methods that include further aspects of the data generating process or observable patterns in the haplotype structure of the data without being prohibitively slow. Some data mining methods have been proposed, e.g. haplotype pattern mining [20] and HapMiner [21], as well as cladistic methods that aim at associating the disease chromosomes to some subset of a reconstructed tree for either the whole or a part of the region surveyed [22-26]. These methods have been applied to small real data sets and have shown to perform well, especially when more than one mutation in the gene is responsible.

We propose to follow a similar approach by building perfect phylogenies around each marker. We accomplish this by using as many markers as possible (on either side of a given marker) that fit on a single phylogenetic tree. We assume the infinite sites model [27], i.e. each segregating site has a unique mutation. Thus, the procedure is equivalent to defining a region around each focal marker, such that all markers in this region are compatible with the marker in focus as judged by the four gamete test [28]. Following, we use different clustering measures of case chromosomes in the phylogeny thus defined. The idea behind the clustering measures is that we treat the perfect phylogeny as a decision tree and measure how well it explains the case/control classification: If the tree explains the classification well, there is an association between the tree topology and the disease, if the tree does not explain the classification, the tree and disease status are considered independent.

## Results

We have implemented our new method, named Blossoc for BLOck aSSOCiation, and evaluated it on a number of simulated datasets and two real datasets, and compared the accuracy with a number of existing methods. Our implementation is efficient enough to handle very large datasets, analysing simulated sets of 10 MB regions of 1000 case and 1000 controls with 1000 SNPs in ~15 seconds on a 3GHz Intel Xeon. Scaling this to 300 K SNPs over the entire genome, the running time will be less than two hours.

**Figure 1:**
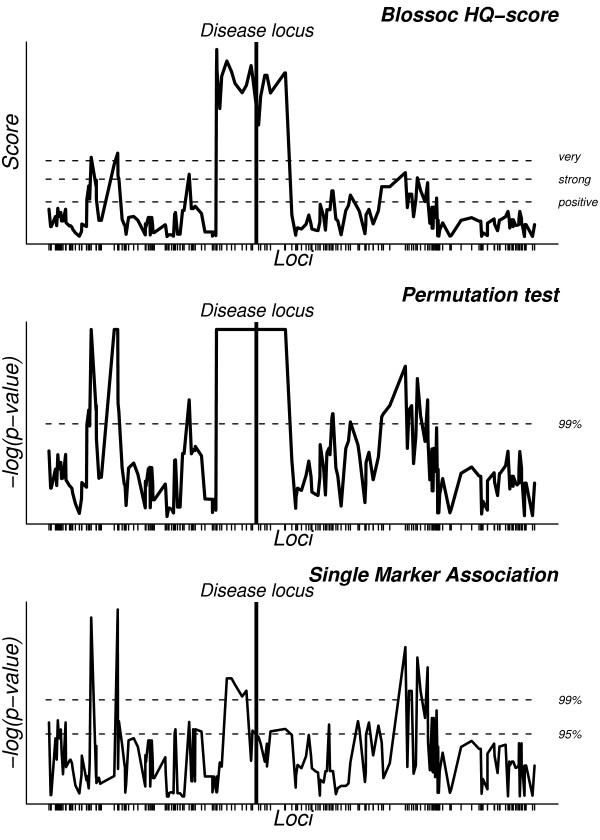
**Example result**. Plot showing the clustering scores from our algorithm for each locus across a region, using the HQC score (top). The horizontal lines indicate "positive" significance, "strong" significance, and "very strong" significance, respectively (see main text). P-values, obtained by a permutation test (1000 replicates), are shown below, where the bottommost line shows 5% significance and the topmost line 1% significance. At the bottom, the corresponding single marker association is shown, where the two lines again show 5% and 1% significance.

In evaluating our algorithm, we calculated scores for each marker locus in a dataset; an example is shown in Fig. 1. Our method does not in any way restrict us to calculating scores for marker loci only, and perhaps the most appropriate approach would be to select the points uniformly placed on the region of interest. However, in our simulated data, the density of markers is high and the marker positions are uniformly randomly placed, so scoring only marker loci is not a major limitation, and has the benefit that we can immediately compare with single marker association (SMA).

Comparing our scoring to SMA, shows a high correlation between high clustering scores for our method and small p-values for SMA. However, our scoring gives a smoother "curve" since neighbouring markers are included when scoring a locus and neighbouring scores are therefore more dependent (compare the topmost and bottommost plots in Fig. 1).

To test significance of scores in our method we generally have to rely on time-consuming permutation tests. Relatively time-consuming tests, that is. Although doing thousands of permutation tests slows down our method significantly, it still completes in around 12 hours for our simulated data sets. Compared to this, many model-based Bayesian approaches requires days of CPU time. When using the BIC score, however, theoretical results in [29], described in the Methods section, suggest to simply use the score to judge evidence of significance. The HQC score is very similar to the BIC score, and hence we would expect to be able to use the HQC score to evaluate significance as well. We tested this suggestion by two different experiments. In the first experiment, we simulated 500 data sets under the null model, i.e. where cases and controls are simulated under the coalescent process, but where status is assigned randomly. This way we collected the null distribution of scores, for which the 95%, 99% and 99.9% percentiles were 0.61, 4.40, and 9.14, respectively.

In the second experiment, we compared scores with p-values obtained by running permutation tests on a subset of our simulated data, selecting 50 random sets with one mutation and 50 random sets with two mutations, each with 1000 individuals (500 cases and 500 controls) and 1000 permutations. Using scores 2, 6, and 10 to mean positive, strong, and very strong evidence for association, as suggested in [29] – and consistent with our null-model simulations – we examined the p-values corresponding to loci with those scores higher, see Table 1. These data indicate that, at least as a good approximation, the scores alone can be used to indicate significant association.

**Table 1:** P-values and HQC scores

Evidence	max p-value	mean p-value (± s.d.)
Positive (HQC > 2)	0.038	0.0021 (± 0.0048)
Strong (HQC > 6)	0.009	0.0003 (± 0.0008)
Very Strong (HQC > 10)	0.004	4.059 · 10^-5 ^(± 0.0002)

Statistics for p-values for different levels of significance as determined by the HQC score.

In the following, we measure the accuracy of our algorithm by taking simply the maximal scoring locus as a point-estimate of the disease locus, and measure the distance from this inferred locus to the true locus. This, of course, does not give as much information about the analysis as does knowledge of the scores over the entire region, but greatly simplifies summarising results over large number of data sets.

### Performance on simulated data sets

**Figure 2:**
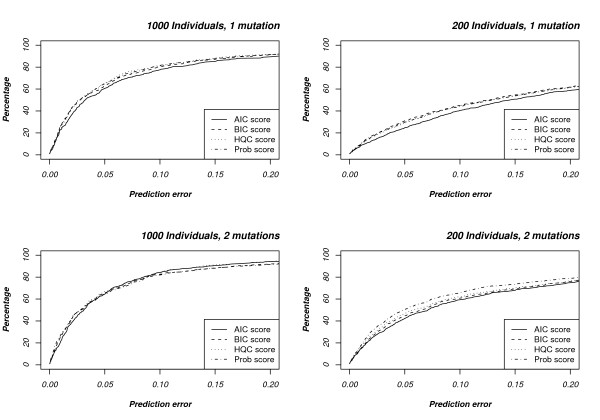
**Comparison of scoring functions**. The x-axis shows the distance from the true locus, in units of *ρ *= 40, i.e. approximately 100 Kbp, while the y-axis shows the accumulated percentage of data sets where the predicted locus falls within a certain distance from the true locus.

We initially investigated the different scoring criteria (AIC, BIC, HQC, and Prob scores, see Methods) on simulated data sets, either haploid or diploid (genotype) data, with a single or two disease mutations, see Fig. 2 and Table 2.

**Table 2:** Score comparison

Setup	AIC score	BIC score	HQC score	Prob. score
1000 individuals				
1 mutation	0.0787 ± 0.118	0.0705 ± 0.111	**0.068 ± 0.111**	**0.068 ± 0.103**
2 mutations	0.061 ± 0.09	0.068 ± 0.113	**0.059 ± 0.091**	0.066 ± 0.103
200 individuals				
1 mutation	0.235 ± 0.230	**0.214 ± 0.227**	0.219 ± 0.227	0.220 ± 0.230
2 mutations	0.148 ± 0.184	0.140 ± 0.179	0.140 ± 0.174	**0.126 ± 0.174**

Summary of the accuracy of the four scoring functions (mean prediction error ± s.d.) in units of *ρ *= 40, i.e. 1.0 corresponds to a recombination rate of *ρ *= 40. For each simulation setup, the best performing score (measured by smallest mean prediction error) is highlighted.

The best scoring function varies from data set to data set, and no scoring scheme is consistently superior to the others, either on individual data sets (Fig. 2) or on average (Table 2). It seems, however, the prob. scoring performs best on smaller datasets while the prob. score and the Hannan and Quinn criterion are about equally accurate on larger datasets. Of the two, however, the HQC is much faster to compute, especially for large datasets, making it our preferred scoring function. As a result of this, we used the HQC score in the following experiments whenever analysing more than 200 individuals and the prob. score whenever analysing fewer. Another experiment (not shown) showed that performance is slightly better when a minimum of 10 markers are forced to be included. It also showed that it did not affect the accuracy significantly if more than 10 markers where forced to be included, because the following pruning (see Methods) removes the additional edges in the tree if they are insigninficant. In all reported experiments, we have therefore required at least 10 markers to be included.

**Figure 3:**
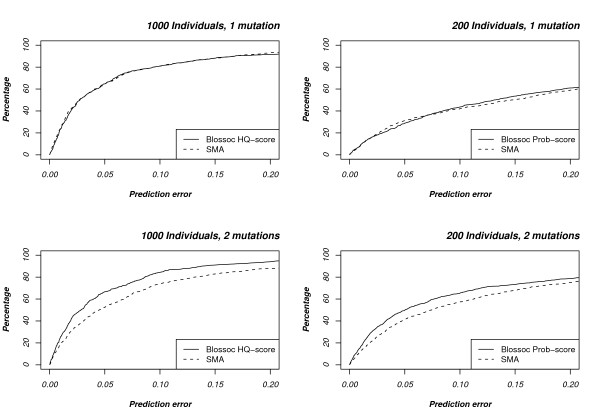
**Comparison with single marker association**. The plots show the accuracy of our method compared with SMA for one and two mutations (topmost and bottommost, respectively) and with 1000 and 200 individuals (leftmost and rightmost, respectively).

Figure 3 compares our method, with these optimal scoring choices, to single marker association. For data sets with a single mutation, the two methods seem equally matched for the large data sets, while the single marker association seems to have a slight advantage on the smaller data sets. For two mutations our method is more accurate than SMA in both cases.

**Figure 4:**
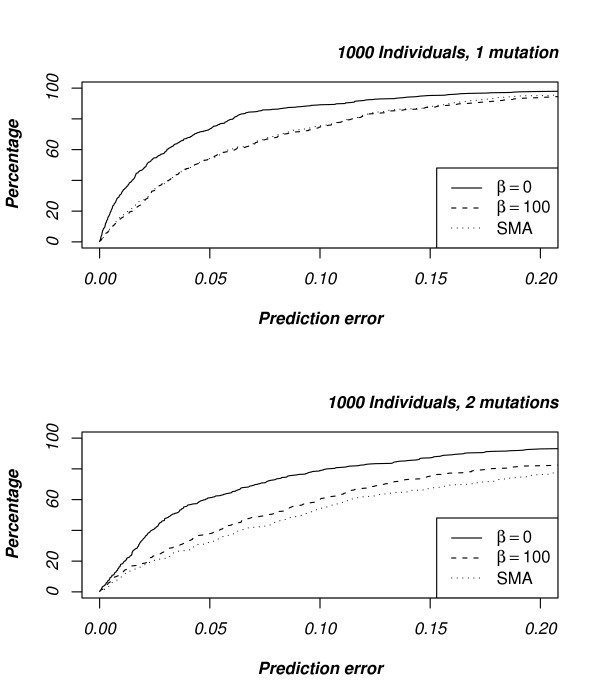
**Robustness under population growth**. The graph compares the mapping accuracy when data was simulated assuming constant population size (*β *= 0) with data simulated assuming exponential growth (*β *= 100). The single marker association accuracy shown is for the exponential growth data sets; for contrast with single marker association without growth, see Fig.3.

The data sets were simulated assuming constant population size, but the human population is believed to have gone through a number of expansions. To test robustness of our method under population growth we also simulated data under exponential growth; growth parameter *β *= 100, see [[30], Chap. 4]. Results are shown in Fig. 4. Encouragingly, the results are not much affected by growth. Compared to data sets without growth, the accuracy for Blossoc drops – which is to be expected since the growth drives the genealogy towards more star-shaped topologies, in which our method will find no signal – but it still behaves similar to single marker association on the one-mutation data sets and it is more accurate on the two-mutations data sets.

**Figure 5:**
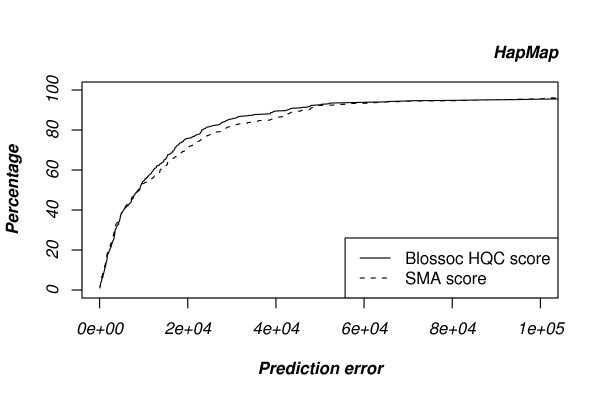
**Performance on data sets generated from HapMap data**. The comparison between out Blossoc method and single marker association resembles the setup for our simulated sequences based on a single mutation.

To avoid systematic bias due to our simulation setup, we also generated data in a completely different manner, by boosting data from the HapMap project (see Methods section). Results for this setup are shown in Fig. 5 and resemble the results obtained from our simulated data.

### Comparison with other methods

The large number of association mapping methods developed makes it infeasible to compare all methods, and thus we are forced to make a choice as to which we should compare our new method to. Our primary criterion in this choice was to compare with methods in the same niche as ours: methods aimed at fast exploration of very large data sets (as opposed to highly accurate at the cost of very long running times). Under this criterion we found the following methods: *HapMiner *[21], *Haplotype Pattern Mining *[20], and *HapCluster *[31]. All three methods are aimed at finding areas in the data where cases appear more similar than controls, but define such areas, and scores loci accordingly, in different ways.

#### Comparison with HapMiner

**Figure 6:**
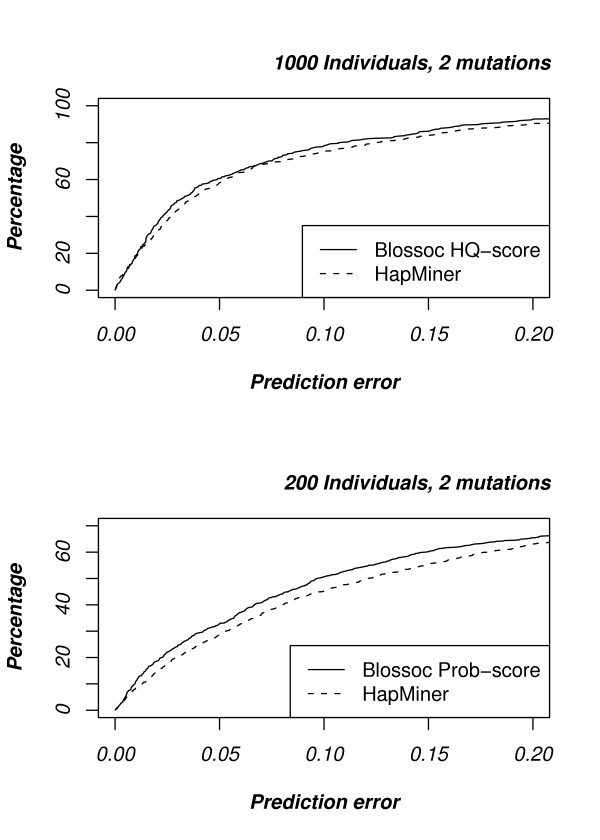
**Comparison with HapMiner**. Topmost plot shows the cumulative error for large data sets while the bottommost plot shows the cumulative error for small data sets, in each case for data sets with two mutations.

An implementation of the HapMiner method is available from the authors' homepage as a binary executable. We downloaded this implementation, and ran it on our simulated data sets with default parameters except for parameter number 5 which was set to 0 to prevent permutation testing. For data sets with a single mutation, whether large or small, the HapMiner method and our method perform similarly (results not shown), but for two mutations Blossoc is slightly more accurate, see Fig. 6.

Compared to our new method, HapMiner is significantly slower, with an average running time of ~40 minutes per data set when run on a 3GHz Intel Xeon, compared to a few seconds for our Blossoc method. The long running times for HapMiner made it impractical to compare the two methods on the larger HapMap based data sets.

#### Comparison with Haplotype Pattern Mining

**Figure 7:**
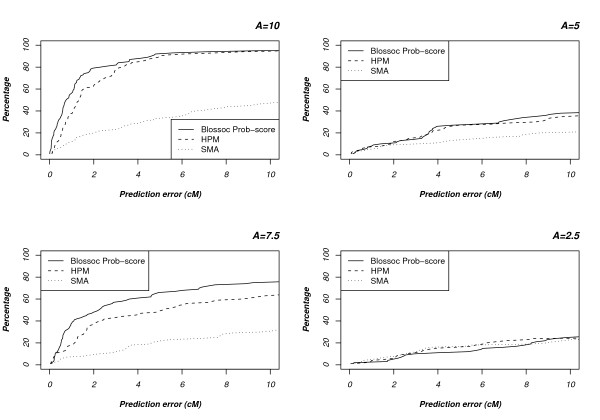
**Comparison with Haplotype Pattern Mining**. The data sets are split into four groups of increasing difficulty, based on the number of cases carrying the disease mutation: *A *= 10%, 7.5%, 5%, and 2.5%.

The Haplotype Pattern Mining (HPM) implementation is not freely available, but the simulated data sets used to evaluate the method in [20] can be obtained from the authors' homepage. We therefore compare the two methods by running Blossoc on these data sets and compare them with the results reported in [20], see Fig. 7. These data sets are much smaller than our own simulated data sets – with 100 cases and 100 controls – so following the guidelines described earlier in this section, we use the prob. score for Blossoc. The data sets come in four different classes, of increasing complexity, based on the number of cases carrying the disease mutation. As shown in Fig. 7, Blossoc is more accurate on the two easiest classes (on the left of the figure) while the two methods are comparable on the two hardest (on the right in the figure). Only on the last data set does single marker association compare to the other two; in the three easiest cases both Blossoc and Haplotype Pattern Mining outperforms single marker association.

#### Comparison with HapCluster

Though an implementation of the HapCluster method is available from the authors, this implementation is in the scripting language R and therefore much too slow to practically compare Blossoc and HapCluster on our simulated data. Instead, as for HPM, we rely on results reported by the authors, and compare Blossoc with HapCluster on the simulated data described in [31]. The data sets in [31] come in five different classes, based on the spacing of markers, the frequency of the causal allele, and the minor allele frequency (MAF) of the markers: All data sets represent regions of 750 Kbp, but the number of SNP markers come in three sizes: 30 (giving an average spacing of 25 Kbp), 75 (a spacing of 10 Kbp) and 150 (a spacing of 5 Kbp); the frequency of the causal allele comes in two ranges: common (15%–25%) and moderately rare (5%–10%); and finally, the MAF was either 5% or 10%. This was combined in five combinations: the 25 Kbp spacing had MAF 10% and common causal allele; the 10 Kbp spacing had MAF 10% and moderately rare causal allele; and the 5 Kbp spacing had two moderately rare causal allele versions, with MAF 5% and 10%, respectively, and one common causal allele version with MAF 10%. All data sets consisted of 200 cases and 200 controls, selected with genotype relative risk in ratio 1:3:9 for the 25 Kbp and 10 Kbp spacing data sets and in the ratio 1:2:4 for the 5 Kbp spacing data sets. Each parameter class was used to simulate 100 data sets.

**Figure 8:**
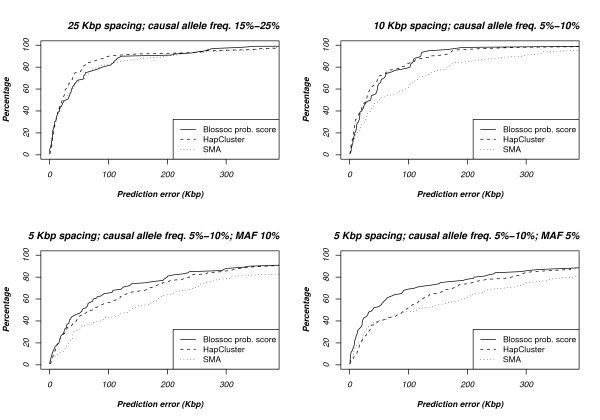
**Comparison with HapCluster**. The plots show the accuracy over 100 data sets for each setup. The data sets are split into four group, depending on the spacing of markers, the allele frequency for the disease allele and the minor allele frequency of the SNP markers.

For the 5 Kbp data set with common causal variant, we saw no difference between Blossoc, HapCluster, and single marker association; a comparison of the three methods is shown for the four other parameter combinations in Fig. 8. For the 25 Kbp spacing data sets, HapCluster seems slightly better than Blossoc and single marker association, while in the 10 Kbp spacing data sets, Blossoc and HapCluster seem equally good and both better than single marker association. For the remaining two data sets, Blossoc is slightly better than the other two.

Since the Blossoc method uses a very simple model of the data to achieve very fast running times – simple tree topologies based on segregating sites only – we don't expect it to be as accurate as more time consuming methods with more detailed data models. Nevertheless, it is interesting to compare our method with such methods to get an impression of how great a loss in accuracy we accept by using the faster method.

Since it is impractical to run CPU intensive methods on our simulated data sets, we choose again to run Blossoc on previously published data sets and compare with only a single, but state of the art, representative of this class of methods, the LATAG method from [25].

We used our Blossoc method to analyse simulated data sets from and compared it with reported results from [25]. The data consist of 50 sets 1 cM regions with 45–65 markers for 30 diploid cases and 30 diploid controls, where the causal allele frequency is in the range 0.1–0.2 and the disease status is assigned with probabilities

*P *(affected | homozygote mutant) = 0.80

*P *(affected | heterozygote) = 0.10

*P *(affected | homozygote wild - type) = 0.05

Compared to the other simulated data sets described previously, these data sets are very small. Complex methods, such as LATAG, do not scale to large data sets – even these very small data sets require hours of CPU time to analyse – but are often capable of detecting signals in data sets where faster methods, such as ours, have insufficient power.

**Figure 9:**
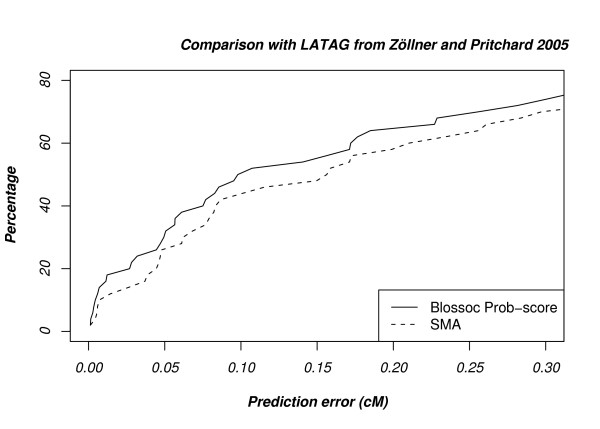
Performance on datasets from [25].

Results are shown in Fig. 9 and Blossoc is again more accurate than SMA. We were not able to get the exact results from Figure 6 in [25], but a visual comparison of Fig. 9 with Figure 6 in [25] suggest that Blossoc is slightly less accurate than LATAG, with an accuracy somewhere half-way between single marker association and the LATAG method. The improved accuracy of LATAG, however, is at the expense of running time where a reported ~5 hours per data set for LATAG needs to be compared with a few seconds per data set for Blossoc.

### Results on real data sets

#### CF Data Set

**Figure 10:**
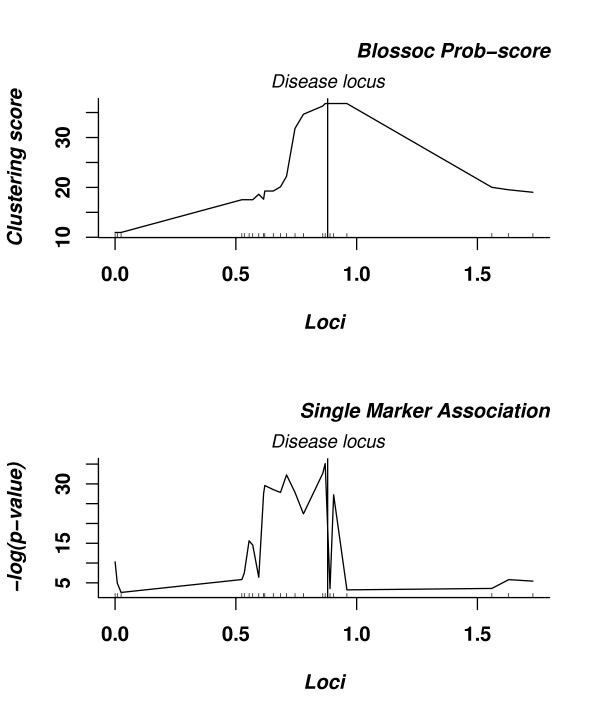
**Analysis of the CF data set**. The topmost plot shows the clustering scores assigned to the loci using our Blossoc method while the bottommost plot shows single marker association.

We ran our method on the ΔF508 mutation for cystic fibrosis data from [32]. The data set consists of 94 cases and 92 controls, genotyped for 23 markers. Because of the small size of the data set we used the prob. score. The results from this analysis are shown in Figure 10. The markers are very un-evenly spaced in the region, and this is a case where a uniform placement of scoring points would probably be preferable to scoring only the marker loci, but to be able to immediately compare it with single marker association (shown at the bottom of the figure) we still use the marker loci. The wide tail to the right of the disease locus, however, is probably an artifact of this choice.

Blossoc takes its maximal score in the true locus, but this maximal score stretches over the range 0.87–0.96 with center at 0.91. This is comparable to other fine-maping tools (Table 3).

**Table 3:** Comparison of location estimates

Method	Estimate	95%-interval
Liu et al. [15]	0.87	0.82–0.93
Morris et al. [14]	0.85	0.65–1.00
Zöllner and Pritchard [25]	0.87	0.81–0.92
Mailund et al. [42]	0.82	0.73–1.03
Blossoc	0.91	NA

Comparison of location estimates of the ΔF508 mutation for cystic fibrosis data [32] by Blossoc and other coalescent-based fine mapping tools. The mutation is located at 0.88. Blossoc only gives a point estimate of the true locus and no credibility interval, but the maximal score stretches over the interval 0.87–0.96.

#### CYP2D6 Data Set

**Figure 11:**
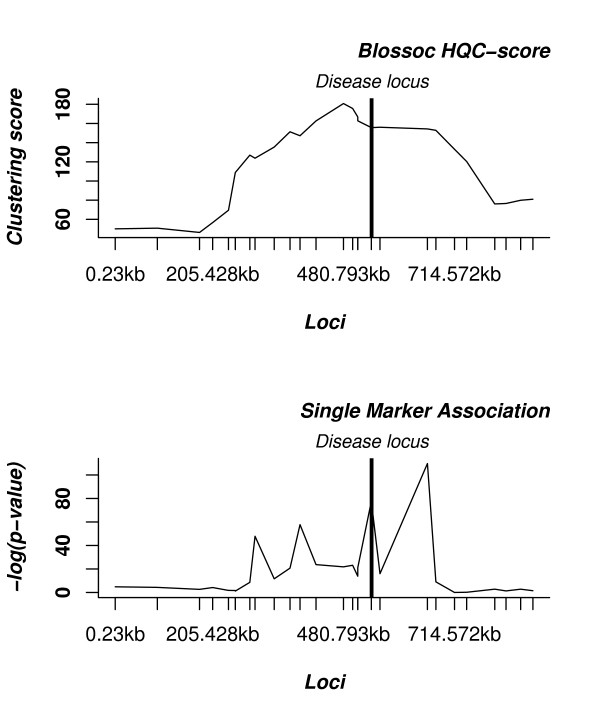
**Analysis of the CYP2D6 data set**. The topmost plot shows the clustering scores assigned to the loci using our Blossoc method while the bottommost plot shows single marker association.

The CYP2D6 gene plays a role in drug metabolism and in [33] 32 SNPs in a 890 kb region spanning the CYP2D6 locus were genotyped in 1,018 individuals, of which 41 were classified as having the "poor drug metaboliser" (PM) phenotype. We ran Blossoc on this dataset and tested for association with the PM phenotype; the results are shown in Fig. 11. The entire region around the CYP2D6 scores very strongly (> 100) with a HQC-score of 156 at the actual gene, but with a higher score, 181, about 60 kb upstream of the gene (but in high LD with the gene).

In [31], Waldron et al. finds two modes in the CYP2D6 dataset when analysed with HapCluster. The smaller of the two corresponds to our maximal peak, while the highest mode of HapCluster is located at the CYP2D6 gene.

## Discussion

Our Blossoc method is based on the very simple idea of compatibility in a region around a marker combined with perfect phylogenies based on binary traits assuming the infinite sites model. This idea has not previously been explicitly exploited for association mapping. A single marker often belongs to different intervals of compatible sets of markers but here we choose the most symmetric interval around the marker in focus (see Methods). An interval with only compatible markers may well have experienced recombination events in the history of a large sample, but these recombination events have left relatively little imprint on the data. Thus, the method uses parts of the observable haplotype structure without making any reference to the underlying evolutionary process that created this haplotype structure – aside from the infinite sites model that is typically considered reasonable for SNP data. Apart from what compatibility tells us about recombination away from a focal marker, the method can be classified as a data mining approach. This can be considered a strength in cases where little is known about the process that generated the data, e.g. the demographic process, the recombination process and the ascertainment biases in choice of markers used.

The Blossoc method is mainly designed to handle a very dense set of markers with high linkage disequilibrium since, in this case, blocks of compatibility are expected to include several markers. To extend the use of the method to cases of more distantly spaced markers we have investigated the effect of forcing a preset minimum number of markers to be included. While this has little effect on densely spaced markers where the number of compatible markers usually exceeds this minimum, it was found to increase the mapping accuracy for less densely spaced markers and to be relatively insensitive to minimum values, as long as they exceed 5, due to the subsequent pruning of the tree.

The method is evidently heuristic and its efficiency can therefore only be evaluated through the application of the method to well defined data sets where the result is already known and comparing its accuracy to other methods. Due to the almost absence of publicly available case-control data sets where susceptibility mutations are known, we have mainly evaluated the performance of the method on artificial data sets either simulated under simple demographic models or based on augmentation of data from the public HapMap project.

We initially used these test data sets to evaluate which scoring function to use for establishing significant clustering, and were able to conclude that the HQC scoring is generally superior except for small data sets (fewer than 100 cases and 100 controls) in which case the prob. score is superior. We thus recommend choice of HCQ if more than 200 sequences are available and otherwise the prob. score. Our experiments also prompt us to recommend that a minimum of 10 markers are always included.

Using our recommended choice of scoring function, the results encouragingly show that the simple method generally outperforms marker by marker association as well as competing fast methods where we could get access to the software and/or previous analyses which we could directly compare our results against. If only a single mutation affects the disease status, then most of the competing methods are indistinguishable from just choosing the most strongly associated single marker. However, when disease heterogeneity or non-additive genotypic effects are modelled, single marker association is inferior to the other methods. Among these, our Blossoc method generally outperforms HapMiner, HPM and HapCluster, even on the data sets used in the original publications of these alternative methods. Blossoc is also very competitive in regards to running time. For our simulated data sets, Blossoc completes the analysis within a few seconds on a 3GHz Xeon processor, compared to less than a second for SMA and ~40 minutes for HapMiner. We do not compare running time with HPM, since we do not have access to the tool, or to HapCluster, since this method has only been implemented as an R prototype, and a runtime comparison with this will not be a fair comparison. Blossoc is thus only slightly slower than the simple single marker association and much faster than HapMiner.

Surprisingly, Blossoc also performs reasonable compared with more involved methods such as the method by Zöllner and Pritchard. However, we expect that model based approaches always will be superior to our method as long as the model is a reasonable approximation to the data generating process, and our experiments also show that Zöllner's and Pritchard's LATAG outperforms Blossoc. Model based approaches, however, are many orders of magnitude slower than our new method, and more importantly will not scale to the size of our own simulated data sets. Blossoc can analyse 3 million SNPs in 1000 cases and 1000 controls in a few days, while the method of Zöllner and Pritchard will not be able to handle such data sets. We expect that boosting the power in a study, by increasing the number of samples and being able to analyse the larger data sets, will more than compensate for the cruder model.

## Conclusion

We have presented a fast method, Blossoc, for accurate localisation of disease causing variants in high density case-control association mapping experiments. Blossoc has the same accuracy as single marker association in the simplest case of a single disease causing mutation and a constant recombination rate. However, when it comes to more complex scenarios of mutation heterogeneity and more complex haplotype structure such as found in the HapMap data our method outperforms SMA as well as other fast, data mining approaches such as HapMiner and Haplotype Pattern Mining (HPM) while being significantly faster.

Several extensions to the method are currently under investigation, e.g. the ability to handle unphased genotype data directly without the pre-phasing step (which at present is more CPU intensive than the actual mapping step), an ability to alleviate gene conversion events by skipping single incompatible markers in the centre of a block of compatible markers, and the ability to handle quantitative traits. Multi-allelic markers such as micro-satellites can already be handled if they are assumed to evolve under the infinite alleles model, but the efficiency of the algorithm has not been investigated in this case. Furthermore, the method is sufficiently fast that investigation of interactions between regions should also be feasible to score by a similar principle [34].

## Methods

### The mapping algorithm

#### The basic algorithm for construcing perfect phylogenies

At a given point in the genome, a sample of cases and controls will be related by a true genealogy that can usually only be partly inferred from a set of markers. Mutations that directly affect the probability of belonging to the case category (disease mutations) induce non-uniform distributions of individuals in sub-parts of the tree: descendants of a given disease mutation will have higher risk of the disease, and all such descendants will be found in some sub-tree rooted in the original mutant. Testing the presence of such a mutant locus therefor reduces to testing for a significant clustering of affected individuals, as has been exploited in several methods [23-25]. The method presented in this paper differs from the methods in [23] and [24] in that we do not restrict ourselves to regions with no or very little recombination, and from [25] in how we infer genealogies.

Assuming an infinite sites model, each polymorphic site splits a present day sample of individual haplotypes into a set of descendants of the original mutant and a set of non-descendants; if a haplotype has a mutant allele, it must be a descendant of the original mutant of which there is only one, and if it is a descendant of a mutant it must also carry the mutant allele at the site. Under this model, assuming no recombinations, the true (un-rooted) genealogy can be inferred, assuming enough segregating sites, and efficient algorithms exist [35]. If there are insufficient segregating sites, the fully resolved tree cannot be reconstructed as some sequences will be indistinguishable and therefore their relationship cannot be inferred. In this case, all resolved edges are still part of the true phylogeny, and the identical sequences will only resolve in high-degree nodes, leading to a multi-furcating tree.

If recombinations have occurred, a single tree may not explain the genealogy of the sample; if the recombination results in trees with different topology on each side of the recombination point – and for large sample sizes, this will be the case in almost all recombinations [30] – then different trees are needed to explain the genealogy for the first part and for the last part of the sample sequences. This can be exploited for locating disease genes. If we can split the sequences into regions where no recombination has occurred, we can infer the trees of these regions and test for significant clustering of affected individuals. The regions containing the significant clusterings are our candidates for containing the disease affecting locus.

Unfortunately, there is no efficient way to recognize the regions without recombinations. Hence we are forced to use heuristics to select the non-recombining regions we build trees over. A sufficient, but not necessary, condition for a recombination to have occurred – assuming the infinite sites model – is the four-gamete test: if all four haplotypes 00, 01, 10, and 11 are observed for two loci, a recombination must have occurred between the two [28]. In the following, we will treat any contiguous set of loci, where no pair of loci has all four haplotypes, as if devoid of recombination events.

**Figure 12:**
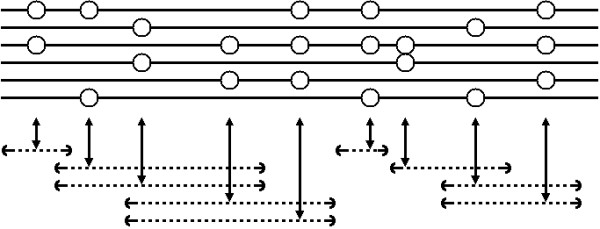
**Compatible regions**. Illustration of the definition of regions around each SNP that are included in building the genealogy. The top-most six solid lines represent chromosomes, where circles represent the presence of a mutant allele and the absence of a circle the presence of the corresponding wild-type allele. The dashed lines show maximal regions around each segregating site, not violating the four-gamete test.

**Figure 13:**
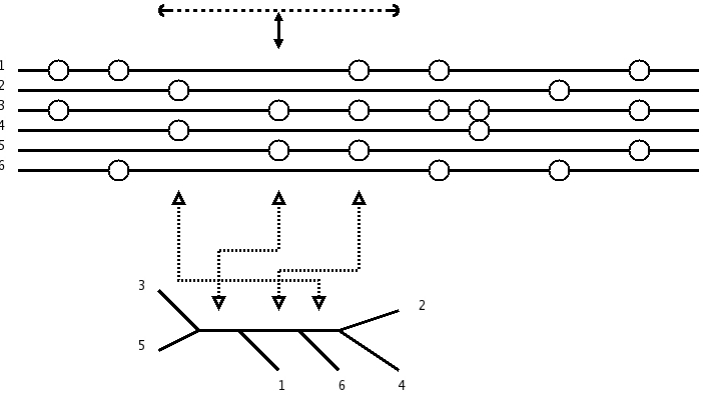
**Local tree**. Local tree for the region of the fourth segregating site. The region covers the third, fourth, and fifth segregating sites. Each site splits the chromosomes into two sets, corresponding to edges in the local tree for site four, shown at the bottom

We attempt to infer the local tree for different loci across the region; for each locus, we consider the region around the locus including as many markers as possible without violating the four-gamete rule, and build the perfect phylogeny for this region, see Figs. 12 and 13. When deciding which markers to include in the perfect phylogeny, we add markers in a "closest to the current marker" ordering, until no new marker can be added without incompatibilities, in one of the directions (left or right), after which we add markers in the other direction until we cannot add markers in that direction either. Selecting regions in this way allow us to use as many neighbouring markers as possible when building the tree of a locus – at the risk of including too many, of course – while attempting to keep the current locus as near center of the region as possible.

In cases where too few markers are included due to many incompatibilities (further described below), incompatible mutations are used to resolve more than one node in the tree; each node that contains sequences with both variants are split into two sub-trees, resulting in a uniquely defined phylogeny.

#### Scoring functions

Once a tree has been built for a locus, we consider the tree a hierarchical clustering of the individuals in the sample, and we perform a test for significant clustering of affected individuals. We define clusters by either inner nodes only containing leaf-subtrees (in which case the cluster consist of all the leaves connected to the node) or single leaves, for leaves not connected to such inner nodes.

If the markers used to build the tree are independent of the affected/unaffected status, we would expect that the affected individuals are distributed identically in all the clusters of the tree; if, on the other hand, the tree is similar to the true genealogy of the disease locus, the affected individuals are expected to be overrepresented in one or more subtrees. The level of significant clustering gives us a score for the tree, and this score is assigned to the locus for which the tree was built.

To score trees, we look at the clustering implied by a tree as a model for the generation of the data, i.e. we consider the perfect phylogeny as a decision tree for the case/control classification, and we assume that the probability of being affected are the same for all individuals that are in the same cluster of the tree, but can vary between clusters. The model selection criteria (MSC) that we used were of the form:

MSC = -2 ln Pr(data|model) + *K *· *D*(*M*)   (1)

Where *K *is the number of free parameters in the model, which is equivalent to the number of clusters in the tree, and *D*(*M*) is a function of the number of samples, *M*, used to penalize over-fitting. The first term is the maximum likelihood estimate and scores how good the model, i.e. the tree, fits the data. The maximum likelihood estimate Pr(data|model) is just factored as the product of the likelihood of each of the individuals. Each cluster has a probability of affectedness independent of the other clusters, and thus the number of free parameters, *K*, is equal to the number of clusters in the tree. The contribution of a cluster *C*, with *C*_*A *_affected individuals, and *C*_*U *_unaffected individuals, to the total log likelihood is:

ln⁡Pr⁡ (C|mod⁡el)=CAln⁡(CACA+CU)+CUln⁡(CUCA+CU)     (2)
 MathType@MTEF@5@5@+=feaafiart1ev1aaatCvAUfKttLearuWrP9MDH5MBPbIqV92AaeXatLxBI9gBaebbnrfifHhDYfgasaacH8akY=wiFfYdH8Gipec8Eeeu0xXdbba9frFj0=OqFfea0dXdd9vqai=hGuQ8kuc9pgc9s8qqaq=dirpe0xb9q8qiLsFr0=vr0=vr0dc8meaabaqaciaacaGaaeqabaqabeGadaaakeaacyGGSbaBcqGGUbGBcyGGqbaucqGGYbGCcqqGGaaicqGGOaakcqWGdbWqcqGG8baFcyGGTbqBcqGGVbWBcqGGKbazcqqGLbqzcqqGSbaBcqGGPaqkcqGH9aqpcqWGdbWqdaWgaaWcbaGaemyqaeeabeaakiGbcYgaSjabc6gaUnaabmaabaWaaSaaaeaacqWGdbWqdaWgaaWcbaGaemyqaeeabeaaaOqaaiabdoeadnaaBaaaleaacqWGbbqqaeqaaOGaey4kaSIaem4qam0aaSbaaSqaaiabdwfavbqabaaaaaGccaGLOaGaayzkaaGaey4kaSIaem4qam0aaSbaaSqaaiabdwfavbqabaGccyGGSbaBcqGGUbGBdaqadaqaamaalaaabaGaem4qam0aaSbaaSqaaiabdwfavbqabaaakeaacqWGdbWqdaWgaaWcbaGaemyqaeeabeaakiabgUcaRiabdoeadnaaBaaaleaacqWGvbqvaeqaaaaaaOGaayjkaiaawMcaaiaaxMaacaWLjaWaaeWaaeaacqaIYaGmaiaawIcacaGLPaaaaaa@6123@

The second term, involving *D*(*M*), penalizes model complexity. The choice *D*(*M*) = 2 gives Akaike's information criterion (AIC) [36], whereas *D*(*M*) = ln(*M*) gives BIC [37], and *D*(*M*) = 2ln ln(*M*) gives the criterion of Hannan and Quinn (HQC) [38].

The penalty in this function is used to avoid over-fitting the data. If there are few incompatibilities the tree for a given position might be large and overfit the data, a well known phenomenon from decision tree literature. A standard way of combating too large trees when building decision trees is to prune any branches where removing the branch improves the score.

We compute the score in a bottom-up fashion, starting at the leaves and pruning any branch that would decrease the trees score.

When building the local tree for a locus we include markers in the order determined by their physical distance from the locus in focus (as described above). By considering the split caused by the first marker the root of the decision tree, this ensures that edges inferred from markers closer to the locus are nearer the root of the tree than edges inferred from markers further away from the locus. This is important for a subsequent pruning since pruning is done from the leaves and moving up in the tree, retains splits caused by SNPs close to the point for which the tree was built, while removing splits caused by SNPs further away (when this improves the score). Keeping edges corresponding to nearby sites higher in the tree than edges caused by sites further away is done solely for technical reasons; it lets us weight nearby markers higher, when pruning, than markers further from the current locus. This should in no way be interpreted as meaning that mutations on nearby markers are more likely to be near the root of the local tree than mutations at other markers. The inferred perfect phylogeny is essentially unrooted, and we have no knowledge of where the "real" root should be placed; we simply select a root that simplifies the decision tree pruning. We never prune the split at the root because this would turn the root into one single cluster with no information about the distribution of affected and unaffected individuals. Since we never remove the root split, if there is no significant information in the tree we just get the single marker score. The smaller the MSC value is, the better the tree explains the data. When scoring the trees we look at the difference between the MSC-value of the tree, *T*_1_, and the MSC value of the simplest tree, *T*_0_, that consists of a single cluster.

ΔMSC=MSC(T0)−MSC(T1)=−2(Ma⋅ln⁡MaM+Mu⋅ln⁡MuM)+2ln⁡Pr (data|T1)−(K−1)⋅D(M)     (3)
 MathType@MTEF@5@5@+=feaafiart1ev1aaatCvAUfKttLearuWrP9MDH5MBPbIqV92AaeXatLxBI9gBaebbnrfifHhDYfgasaacH8akY=wiFfYdH8Gipec8Eeeu0xXdbba9frFj0=OqFfea0dXdd9vqai=hGuQ8kuc9pgc9s8qqaq=dirpe0xb9q8qiLsFr0=vr0=vr0dc8meaabaqaciaacaGaaeqabaqabeGadaaakeaafaqaaeWadaaabaGaeuiLdqKaeeyta0Kaee4uamLaee4qameabaGaeyypa0dabaGaeeyta0Kaee4uamLaee4qamKaeiikaGIaemivaq1aaSbaaSqaaiabicdaWaqabaGccqGGPaqkcqGHsislcqqGnbqtcqqGtbWucqqGdbWqcqGGOaakcqWGubavdaWgaaWcbaGaeGymaedabeaakiabcMcaPaqaaaqaaiabg2da9aqaaiabgkHiTiabikdaYmaabmaabaGaemyta00aaSbaaSqaaiabdggaHbqabaGccqGHflY1cyGGSbaBcqGGUbGBdaWcaaqaaiabd2eannaaBaaaleaacqWGHbqyaeqaaaGcbaGaemyta0eaaiabgUcaRiabd2eannaaBaaaleaacqWG1bqDaeqaaOGaeyyXICTagiiBaWMaeiOBa42aaSaaaeaacqWGnbqtdaWgaaWcbaGaemyDauhabeaaaOqaaiabd2eanbaaaiaawIcacaGLPaaaaeaaaeaaaeaacqGHRaWkcqaIYaGmcyGGSbaBcqGGUbGBcqqGqbaucqqGYbGCcqqGGaaicqGGOaakcqqGKbazcqqGHbqycqqG0baDcqqGHbqycqGG8baFcqqGubavdaWgaaWcbaGaeGymaedabeaakiabcMcaPiabgkHiTiabcIcaOiabdUealjabgkHiTiabigdaXiabcMcaPiabgwSixlabdseaejabcIcaOiabd2eanjabcMcaPaaacaWLjaGaaCzcamaabmaabaGaeG4mamdacaGLOaGaayzkaaaaaa@8148@

Where *M*_*a *_and *M*_*u *_is the number of affected and unaffected individuals, respectively, in the entire data set. According to [29] the ΔMSC value is approximately equal to two times the logarithm of the Bayes factor if the BIC model selection criterion is used. This means that it can be used directly as a measure of significance. Since the HQ model selection criterion approximates the same value as the BIC, this correspondence between ΔBIC and significance should also apply to the HQ criterion. This claim is supported by empirical tests shown in the results section.

Alternatively, statistical significance of a given score is evaluated by a permutation test where the case-control status of all haplotypes is randomly permuted and the tree is pruned again and the score is recalculated. The P-value is then the fraction of permutations where the score is larger than the score obtained from the original data set.

We have also tried another scoring method for the trees, a method that is not based on the model selection criterion. In this method we give scores to trees by looking only at the subtree that has the most significant over-representation of affected individuals. When scoring a cluster we calculate the probability that the cluster would get a number of affected individuals that is greater than or equal to its present number, if the affected/unaffected status of all the individuals were reassigned at random. This probability is small if there is a significant over-representation of affected individuals in a cluster. The probability that a subtree consisting of *n *individuals has got at least *m *affected individuals is:

ps=∑i=mn(ni)⋅p(a)i⋅p(u)n−i     (4)
 MathType@MTEF@5@5@+=feaafiart1ev1aaatCvAUfKttLearuWrP9MDH5MBPbIqV92AaeXatLxBI9gBaebbnrfifHhDYfgasaacH8akY=wiFfYdH8Gipec8Eeeu0xXdbba9frFj0=OqFfea0dXdd9vqai=hGuQ8kuc9pgc9s8qqaq=dirpe0xb9q8qiLsFr0=vr0=vr0dc8meaabaqaciaacaGaaeqabaqabeGadaaakeaacqWGWbaCdaWgaaWcbaGaem4Camhabeaakiabg2da9maaqahabaWaaeWaaeaafaqabeGabaaabaGaemOBa4gabaGaemyAaKgaaaGaayjkaiaawMcaaiabgwSixJqaciab=bhaWjabcIcaOiabdggaHjabcMcaPmaaCaaaleqabaGaemyAaKgaaOGaeyyXICTaemiCaaNaeiikaGIaemyDauNaeiykaKYaaWbaaSqabeaacqWGUbGBcqGHsislcqWGPbqAaaaabaGae8xAaKMaeyypa0JaemyBa0gabaGaemOBa4ganiabggHiLdGccaWLjaGaaCzcamaabmaabaGaeGinaqdacaGLOaGaayzkaaaaaa@533A@

Where *p*(*a*) and *p*(*u*) is the fraction of affected and unaffected individuals, respectively, in the entire data set the score of a tree, *T*, is made from the subtree with the smallest p-value:

Score(T)=min⁡s∈subtree(T)(−ln⁡ps+depth(s))     (5)
 MathType@MTEF@5@5@+=feaafiart1ev1aaatCvAUfKttLearuWrP9MDH5MBPbIqV92AaeXatLxBI9gBaebbnrfifHhDYfgasaacH8akY=wiFfYdH8Gipec8Eeeu0xXdbba9frFj0=OqFfea0dXdd9vqai=hGuQ8kuc9pgc9s8qqaq=dirpe0xb9q8qiLsFr0=vr0=vr0dc8meaabaqaciaacaGaaeqabaqabeGadaaakeaacqqGtbWucqqGJbWycqqGVbWBcqqGYbGCcqqGLbqzcqGGOaakcqWGubavcqGGPaqkcqGH9aqpdaWfqaqaaiGbc2gaTjabcMgaPjabc6gaUbWcbaGaem4CamNaeyicI4Saee4CamNaeeyDauNaeeOyaiMaeeiDaqNaeeOCaiNaeeyzauMaeeyzauMaeiikaGIaemivaqLaeiykaKcabeaakmaabmaabaGaeyOeI0IagiiBaWMaeiOBa4MaemiCaa3aaSbaaSqaaiabdohaZbqabaGccqGHRaWkcqqGKbazcqqGLbqzcqqGWbaCcqqG0baDcqqGObaAcqGGOaakcqWGZbWCcqGGPaqkaiaawIcacaGLPaaacaWLjaGaaCzcamaabmaabaGaeGynaudacaGLOaGaayzkaaaaaa@61EF@

Where depth(*s*) is the depth of the subtree *s *in the tree ie. the number of markers necessary to determine the subtree. In the results section we refer to this scoring function as the Prob score.

**Figure 14:**
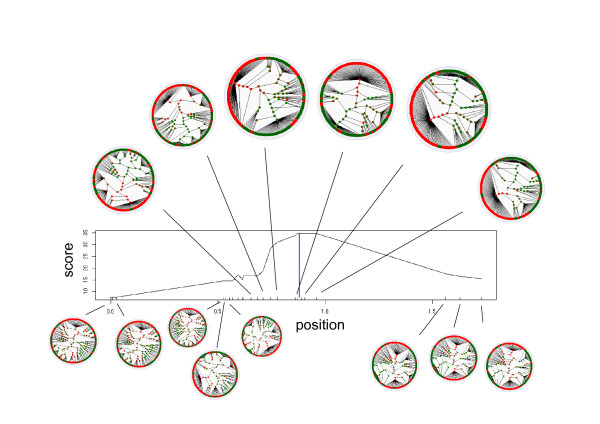
**Local trees for the CF dataset**. Illustration of the decision trees and scores of markers (Prob-score) for the cystic fibrosis data set. In each circle diagram, the case leaves are red and the control leaves green. The branching patterns are defined by the markers included in the segment around the marker in focus, which is also placing the root in the decision tree. The decision trees shown are before pruning is attempted. Pruning collapses som of the branch points. The score is then calculated and can be seen to attain a maximum close the the true location of the the causative mutation (vertical line). The decision trees can also be visually seen to cluster cases more around the causative variant.

Figure 14 illustrates the definition of decicion trees and their associated scores for the 23 markers in the cystic fibrosis data set.

#### Data with many incompatibilities

With a large sample size, the width of regions where all markers are compatible tends to be very small. Consequently, the markers that can be used to split sampled individuals into clusters are few and the resulting trees will contain only few edges; in the limit, only the single marker for which the tree is built will be used, reducing the method to a single marker association test. To alleviate this problem, we forced a minimum number of markers or a minimum length of sequence to be included. In this case the markers in a region can be incompatible, so we cannot build a perfect phylogeny under the infinite sites model. Instead, when adding a marker to the tree that is incompatible with one or more of the markers already added, we add as many new splits as needed to separate individuals with the 0 allele at the new marker from individuals with the 1 allele in the tree.

This heuristic approach to dealing with many incompatibilities is clearly not justified by the infinite sites model with recombination; under this model, the resulting tree is clearly a combination of two or more local phylogenies, since the incompatibility implies that at least one recombination has occurred. The splits in the tree, however, still provide information about the local genealogy, and the reasoning behind the heuristic is that this information is not completely lost when combining two or more local trees, as long as the (unknown) number of trees is low. Simulation results (now shown) justifies this, since using the heuristic outperforms relying on single marker association for the regions where no tree can be built using only compatible markers.

### Tests of the algorithm

#### Simulated data sets

We simulated data sets with a known causative locus with one or two responsible mutations using the coalescent based simulator CoaSim [39]. For each data set, we simulated 20,000 (haploid) sequences and paired them up randomly to obtain (phased) genotype data sets. The sequences were simulated with scaled recombination rate *ρ *= 40-corresponding to 0.1 centi-Morgan assuming an effective population size of 10,000, or roughly 100 Kbp assuming 1 cM = 1 Mbp – and with 200 SNP markers placed uniformly at random over the sequence.

For a fixed simulated ancestral recombination graph, rejection sampling was used to ensure that all minor allele frequencies of the SNP markers were above 10%, reflecting a typical SNP selection bias. The causative locus was placed uniformly at random on the simulated sequence, and we simulated either one or two mutations on the locus, rejecting the entire simulation when the mutant allele frequency was outside the range 18%–22%, for the single mutation case, or outside the range 9%–11% for both mutations in the two mutation cases. We assigned affected status using probabilities:

*P *(affected | homozygote mutant) = 0.20

*P *(affected | heterozygote) = 0.08

*P *(affected | homozygote wild - type) = 0.05

After assigning affected/unaffected status to the simulated individuals, we randomly selected an equal number of affected and unaffected as our test data set. From the simulated genotypes we made data sets of two sizes; large data sets where we selected 500 affected and 500 unaffected individuals (or 1000 haploid sequences of each class) and small data sets where we selected 100 affected and 100 unaffected individuals (or 200 haploid sequences of each class).

#### Artificial data sets created from HapMap

A random 1 Mb section (chromosome 3, position 64893548–65893547, containing 1646 SNPs) of the HapMap data from the Chinese and Japanese populations (90 independent individuals in total) were downloaded from the HapMap home page [40]. For each of the 90 unphased genotypes (with missing data) 50 different genotype configurations without missing data were sampled using fastPHASE [41] resulting in a data set with 4500 genotypes. The sampling utilises that fastPHASE estimates probabilities of all possible haplotypes consistent with the sampled genotypes, and additional haplotypes were sampled using these probabilities. From this data set a marker with minor allele frequency in the range 20%–30% was subsequently treated as causing a disease imitation. The genotypes in the sample were given status as case or control based on the value at the marker with probabilities:

*P *(affected | homozygote mutant) = 0.30

*P *(affected | heterozygote) = 0.15

*P *(affected | homozygote wild - type) = 0.075

Next the data set was created by sampling 500 cases and 500 controls from the sample and 400 of the markers whereupon analyses were performed using our algorithm and SMA. After determining disease status for the simulated individuals, the marker at the selected disease locus was removed. This procedure was repeated to make 500 different data sets.

#### Tests on real data sets

To test our method on real data, we used the CF data set described in [32] the CYP2D6 data set described in [33].

## Availability and requirements

**Project name**: Blossoc

**Project home page**: 

**Operating system(s)**: Binaries available for Intel Linux; should compile on all platforms with C++ and Qt 3 for the GUI version.

**Programming language**: C++

**Licence**: GNU GPL

## Authors' contributions

TM conceived the original approach and all authors participated in developing it. TM and SB implemented the method in the software package Blossoc and SB conducted the experiments. All authors participated in drafting the manuscript.
